# The Type 1 Diabetes T Cell Receptor and B Cell Receptor Repository in the AIRR Data Commons: a practical guide for access, use and contributions through the Type 1 Diabetes AIRR Consortium

**DOI:** 10.1007/s00125-024-06298-y

**Published:** 2024-10-29

**Authors:** Stephanie J. Hanna, Rachel H. Bonami, Brian Corrie, Monica Westley, Amanda L. Posgai, Eline T. Luning Prak, Felix Breden, Aaron W. Michels, Todd M. Brusko, Type 1 Diabetes AIRR Consortium, Erin Baschal, Karen Cerosaletti, Lorissa Corrie, Iria Gomez-Tourino, Lauren Higdon, Sally C. Kent, Peter Linsley, Maki Nakayama, Kira Neller, William E. Ruff, Luc Teyton

**Affiliations:** 1 Division of Infection and Immunity, Cardiff University School of Medicine, Cardiff, UK; 2 Department of Medicine, Division of Rheumatology and Immunology, Vanderbilt University Medical Center, Nashville, TN USA; 3 Department of Pathology, Microbiology, and Immunology, Vanderbilt University Medical Center, Nashville, TN USA; 4 Vanderbilt Center for Immunobiology, Nashville, TN USA; 5 Vanderbilt Institute for Infection, Immunology, and Inflammation, Nashville, TN USA; 6 Department of Biological Sciences, Simon Fraser University, Burnaby, BC Canada; 7 iReceptor Genomic Services, Summerland, BC Canada; 8 the(sugar)science, Redondo Beach, CA USA; 9 Department of Pathology, Immunology, and Laboratory Medicine, College of Medicine, Diabetes Institute, University of Florida, Gainesville, FL USA; 10 Department of Pathology and Laboratory Medicine, Perelman School of Medicine, University of Pennsylvania, Philadelphia, PA USA; 11 Barbara Davis Center for Diabetes, University of Colorado School of Medicine, Aurora, CO USA; 12 Department of Pediatrics, College of Medicine, Diabetes Institute, University of Florida, Gainesville, FL USA; 13 Department of Biochemistry and Molecular Biology, College of Medicine, Diabetes Institute, University of Florida, Gainesville, FL USA

**Keywords:** AIRR, AIRR Data Commons, Autoantibodies, B cell receptors, FAIR data, Next-generation sequencing, Single-cell RNA-seq, T cell receptors, Type 1 diabetes

## Abstract

Human molecular genetics has brought incredible insights into the variants that confer risk for the development of tissue-specific autoimmune diseases, including type 1 diabetes. The hallmark cell-mediated immune destruction that is characteristic of type 1 diabetes is closely linked with risk conferred by the HLA class II gene locus, in combination with a broad array of additional candidate genes influencing islet-resident beta cells within the pancreas, as well as function, phenotype and trafficking of immune cells to tissues. In addition to the well-studied germline SNP variants, there are critical contributions conferred by T cell receptor (TCR) and B cell receptor (BCR) genes that undergo somatic recombination to yield the Adaptive Immune Receptor Repertoire (AIRR) responsible for autoimmunity in type 1 diabetes. We therefore created the T1D TCR/BCR Repository (The Type 1 Diabetes T Cell Receptor and B Cell Receptor Repository) to study these highly variable and dynamic gene rearrangements. In addition to processed TCR and BCR sequences, the T1D TCR/BCR Repository includes detailed metadata (e.g. participant demographics, disease-associated parameters and tissue type). We introduce the Type 1 Diabetes AIRR Consortium goals and outline methods to use and deposit data to this comprehensive repository. Our ultimate goal is to facilitate research community access to rich, carefully annotated immune AIRR datasets to enable new scientific inquiry and insight into the natural history and pathogenesis of type 1 diabetes.

**Graphical Abstract:**

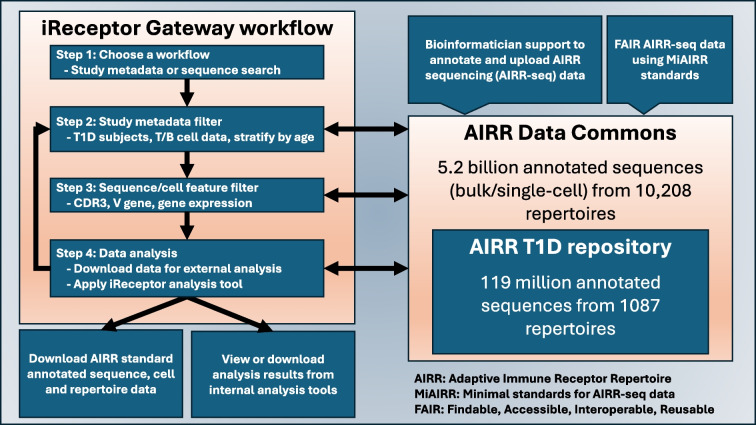

## Introduction

Recombination of variable (V), diversity (D) and joining (J) gene segments within the T cell receptor (TCR) and B cell receptor (BCR) loci forms a diverse array of antigen receptors that are known as the Adaptive Immune Receptor Repertoire (AIRR). Repertoire diversity is essential for pathogen defence, and recent advances in AIRR sequencing (AIRR-seq) technology and machine learning (ML) analyses have enabled the identification of disease-specific AIRR signatures providing high sensitivity and specificity for certain viral infections [1, 2]. These successes have prompted intense interest in the AIRR as a potential means to improve prediction and monitor the progression of autoimmune diseases, such as type 1 diabetes.

In this methods paper, we first overview the current state of the science of TCR and BCR repertoire studies in human type 1 diabetes, and the specific challenges of developing analytical approaches to harness specific repertoire attributes as biomarkers. We then summarise existing frameworks to access type 1 diabetes AIRR-seq data and their respective limitations. The iReceptor platform [3] and AIRR Data Commons (ADC) [4] will address the needs currently facing the type 1 diabetes research community, as evidenced by how they have been used to share AIRR-seq data in other conditions, including cancer and infectious diseases, as detailed further below. We overview how to upload datasets to share with the community and provide use case examples to demonstrate how the available data may be best leveraged. Finally, we look ahead to future developments and collaborations within the type 1 diabetes field.

## Current type 1 diabetes biomarkers to predict disease risk and track progression toward diabetes

Several islet autoantibody (AAb) screening programmes have been developed that predict diabetes onset. For example, Type 1 Diabetes TrialNet screens first-degree relatives of individuals with type 1 diabetes for IAA and GADA, followed by IA-2A, ICA and ZnT8A for those positive in the first screening. These islet AAb predict type 1 diabetes risk and stage disease progression: seropositivity for two or more of these islet AAb (≥2AAb+, stage 1 type 1 diabetes) confers ~70% risk of developing diabetes within 10 years, and a nearly 100% lifetime risk [5–7]. Predicted time to diabetes onset can be further refined by considering which islet AAb are positive; ZnT8A positivity most typically appeared after other islet AAb were detected [8], and was associated with increased risk of developing diabetes on follow-up [6, 8]. Multiple studies, summarised in a systematic review, have demonstrated that IA-2A positivity is particularly associated with disease progression in children [6]. The Environmental Determinants of Diabetes in the Young (TEDDY) study data suggest that very young children are more likely to have IAA as the first AAb, with older children more likely to become GADA positive first [9]. IAA and IA-2A titres are associated with higher type 1 diabetes risk and more rapid progression [6].

Furthermore, type 1 diabetes staging provides a convenient, albeit imperfect, framework to categorise participants, based on evidence of islet autoimmunity and extent of dysglycaemia. Stage 1 type 1 diabetes is defined as the presence of multiple islet AAbs and normal glucose measures. In individuals with ≥2AAb+, impaired glycaemic control, measured through an OGTT to assess beta cell function, defines progression to stage 2 as outlined in Table 1 [7, 10]. Stage 3 is defined as onset of diabetes and stage 4 describes individuals with established diabetes. Other metrics have been proposed, such as continuous glucose monitoring (CGM) data [11] or Index60 (i.e. a composite measure of blood glucose and C-peptide levels across OGTT time points) [12]. However, all of these metrics are imperfect, as islet AAb positivity and OGTT results can vary over time [13]. Even in recently diagnosed stage 3 type 1 diabetes, variations in OGTT results and other metrics make tracking disease progression and success/failure of immunotherapies problematic [14, 15]. Furthermore, these studies have made clear that, when relying on C-peptide measurements as the primary outcome, trials require large numbers of participants and often take 12 months or longer to confirm a positive response to immunotherapy [15]. Genetic risk scores (GRS) and polygenic risk scores (PRS) also support diabetes onset prediction (reviewed in [16]). A combined risk score (CRS), which incorporated genetic, clinical and immunological factors, improved diabetes onset prediction over AAb data alone [17].

**Table 1:** Type 1 diabetes disease staging criteria

	Stage 1	Stage 2	Stage 3^a^
≥2 islet AAb	Yes	Yes	Yes
Glucose tolerance classification^b^	Normal	Impaired	Diabetes
Fasting OGTT blood glucose, mmol/l (mg/dl)	<6.1 (110)	≥6.1 (110), <7.0 (126)	≥7.0 (126)
30, 60 or 90 min OGTT blood glucose, mmol/l (mg/dl)	<11.1 (200)	≥11.1 (200)	NA
2h OGTT blood glucose, mmol/l (mg/dl)	<7.8 (140)	≥7.8 (140), <11.1 (200)	≥11.1 (200)
Symptoms	No	No	Yes^c^

Staging criteria are in alignment with Insel et al 2015 [7]

^a^Type 1 diabetes diagnosis (stage 3) made if: (1) any OGTT time points are abnormal; (2) HbA_1c_ is ≥48 mmol/mol (6.5%); or (3) casual blood glucose ≥11.1 mmol/l (200 mg/dl) and hyperglycaemia symptoms are present

^b^75 g OGTT: two consecutive normal (stage 1) or impaired (stage 2) OGTT tests are required for these classifications. Impaired glucose tolerance can also be defined as HbA_1c_ ≥39 mmol/mol (5.7%) but <48 mmol/mol (6.5%)

^c^The presence of symptoms may vary

## Immune repertoire analysis can predict disease progression/severity or pathogen exposure in autoimmunity, cancer and infectious disease

Immune repertoire profiling has potential advantages over current risk stratification assays for type 1 diabetes. With respect to antibody profiling, serological detection of secreted antibodies reflects a state wherein B cell tolerance is already broken. Some of the antigen targets are intracellular, implying that destruction of pancreatic beta cells is underway. Furthermore, antibody-forming cells are at a mature stage of B cell development. In the case of BCR gene rearrangement analysis, it may be possible to detect B cell clones with pathogenic potential at earlier stages of maturation, which could provide a means of detecting disease risk at an earlier stage, including the initial emergence of autoimmunity. For TCR analysis, there are already early hints that this type of stratification is possible. For example, analysis of high-throughput TCR β chain (TCRβ) repertoire data showed that anti-insulin T cell clones increased in at-risk individuals who progressed to diabetes relative to those who did not develop diabetes over the same time period [18]. In contrast, clonal changes in influenza A-reactive T cells were not observed in this same cohort [18]. The concept that the repertoire landscape can predict immunological exposures is already well entrenched for infectious diseases and it is not a huge stretch to think that, with adequate databases, similar disease-associated repertoire motifs could be unearthed in type 1 diabetes. With respect to infectious disease, TCR sequencing technology was used to discriminate cytomegalovirus (CMV) seropositive from seronegative individuals with high sensitivity and specificity [1]. An ML approach discriminated between severe acute respiratory syndrome coronavirus 2 (SARS-CoV-2)-infected vs SARS-CoV-2-naive individuals based on TCR sequencing data [19]. As another example, DeepTCR was used with the ImmuneCODE database to identify TCR sequence motifs associated with recognition of specific SARS-CoV-2 epitopes that tracked increased illness severity [20, 21].

Immune repertoire profiling also has advantages over genetic markers of disease progression in type 1 diabetes. This advantage stems from the fact that BCR and TCR repertoires are influenced not only by inherited differences (e.g. germline allele usage, duplications and deletions, HLA haplotypes) but also by somatic events (recombination and, specifically in the case of BCRs, somatic hypermutation and class switching) and immune selection (e.g. clonal diversification, expansion and contraction). These somatic and selection events provide an opportunity to link immune exposures to disease and monitor clones over time to evaluate the effects of therapy. These concepts are already familiar in haematological malignancies, where clonal BCR heavy chain sequences can be used to monitor B cell acute lymphoblastic leukaemia patients for evidence of minimal residual disease [22]. Furthermore, TCR sequencing of tumour-infiltrating T cells can be used to identify cancer-related TCRs that can be tracked based on their distinctive third complementarity determining region (CDR3) sequences into the circulation and used to differentiate individuals with cancer from healthy control individuals [23]. In autoimmunity, single-cell BCR sequencing identified autoantigen-specific B cells in individuals with the neuromuscular autoimmune disease, myasthenia gravis. These B cell clones persisted in spite of B cell depletion therapy, raising the possibility that clone tracking can be used to predict which patients will respond or fail to respond to certain forms of immunomodulatory therapy [24].

## Heterogeneous individual responses to type 1 diabetes immunotherapies complicate clinical trial enrolment and evaluation

A number of immunotherapies targeting B and T cells are in development for type 1 diabetes. Of these, a single course of teplizumab (an anti-CD3 monoclonal antibody) provided a 2.5 year delay in clinical diabetes onset compared with placebo [25, 26], and is now approved by the US Food and Drug Administration (FDA) for use in stage 2 type 1 diabetes. Other experimental T cell immunotherapies include abatacept (cytotoxic T-lymphocyte associated protein 4 [CTLA4]-Ig), which limits T cell activation by blocking CD28 interaction with costimulatory molecules and preserves C-peptide levels in individuals with recent-onset type 1 diabetes [27], and anti-thymocyte globulin (ATG) [28, 29], which increases the ratio of CD4^+^ regulatory T cells (Treg) to CD4^+^ conventional T cells (Tconv) and promotes CD4^+^ T cell exhaustion. B cells have also been targeted: rituximab (anti-CD20) transiently preserved C-peptide in people with recent-onset type 1 diabetes [30]. However, despite the promise of these and other immunotherapies, challenges remain.

A common theme in all type 1 diabetes immunotherapy trials to date is heterogeneity in individual responses. Several studies have attempted to address this heterogeneity by looking at changes in specific populations of immune cells in responder vs non-responder groups. For example, islet-autoreactive T cells with a distinct cytokine secretion pattern were linked to response to alefacept [31]. Additionally, T follicular helper (Tfh) cell profiles predicted abatacept response, with baseline Tfh-like cell frequencies differentiating responder and non-responder groups [32]. However, approaches that focus on changes in phenotypic subsets of lymphocytes can suffer from a signal-to-noise ratio problem. As an example, the Tfh compartment may contain autoreactive T cell clones, but may also contain T cell clones elicited by protective immune responses, particularly following vaccination or infection. T cell effector function, in addition to autoantigen reactivity, also bears consideration. For example, the overall frequency of insulin-reactive T cells was not different between individuals with and without type 1 diabetes, but insulin peptide responses skewed towards proinflammatory (IFN-ɣ) and away from regulatory (IL-10) cytokine production among cells isolated from donors with type 1 diabetes relative to those present in healthy control donors [33]. Developing molecular-based approaches to focus on the signal (islet-autoreactive T and B cell clones and their phenotypes) rather than the noise (T and B cell clones unrelated to type 1 diabetes) hold potential to further augment evaluation of experimental and approved immunotherapies in type 1 diabetes. Clinical trial endpoints commonly focus on changes in beta cell function, which is inherently downstream of autoimmune destruction of beta cells. Developing approaches to monitor mechanistic immune changes that track with drug efficacy could accelerate clinical trial evaluation over traditional endpoints that require waiting for beta cell damage (or preservation) to become apparent.

## State of the science of AIRR-seq data and type 1 diabetes

Until recently, AIRR-seq datasets in type 1 diabetes were relatively few in number, particularly those examining paired TCRα and TCRβ or BCR heavy and light chain sequences, which are key to truly understanding receptor specificity for antigens (reviewed in [34]). However, with the widespread adoption of single-cell RNA-seq (scRNA-seq) and progress in next-generation sequencing (NGS) of TCRs and BCRs, the available datasets have blossomed (representative examples in [18, 31, 35, 36]). Still, there are difficulties with applying AIRR-seq analysis to type 1 diabetes research. A paucity of ‘public’ type 1 diabetes autoantigen-specific TCRs (i.e. autoantigen-specific TCRs widely shared between people with type 1 diabetes) makes modelling the type 1 diabetes immune response particularly challenging [37, 38]. There is some evidence for shared TCRα chains [39], whilst a recent paper demonstrated a number of public TCRβ chains in type 1 diabetes [40]. Diabetes autoantigen-specific TCRs have also been identified using different methods (reviewed in [41]). At the most stringent level, this has involved re-expressing the TCR in a cell-based system to identify the peptide epitopes recognised [38]. Useful datasets have also been generated through other assays developed to measure islet-specific immune responses (reviewed in [42]). These include in vitro stimulation assays (commonly known as activation-induced marker [AIM] assays [43, 44]), tetramer/multimer sorting from peripheral blood [45], or sequencing of TCR repertoires from the pancreas and pancreatic lymph nodes (pLNs) of organ donors with type 1 diabetes, which are likely to be enriched in diabetes autoantigen-specific TCRs [41]. However, it is expected that all of these repertoires will also include non-specific TCRs, for example from non-specific binding to multimers, by-stander activation in AIM assays, or incidental location of T cells in the pancreas and pLNs. Furthermore, other datasets of TCR sequences and T cell gene expression from participants in longitudinal studies of type 1 diabetes development [18, 46], and clinical trials of immunotherapies in type 1 diabetes, are also emerging [42]. These may include important information on the overall TCR repertoire dynamics and features in people with and at risk for type 1 diabetes; for example, shorter CDR3 length has been observed in people with type 1 diabetes [47]. Nevertheless, integrating these different datasets presents many challenges. Key to the success of this project will be the ability to make the data available in a standardised but accessible format with all possible metadata included. ML approaches offer the tantalising possibility of being able to predict antigen specificity from TCR sequence and HLA typing [41, 48]. Finally, surveying lymphocytes in peripheral blood or formalin-fixed tissues has created a wealth of data, but these data do not fully capture the impact of the inflammatory milieu and dynamic nature of lymphocyte interactions in tissues. Pancreas slice technology may help to address some of these challenges by enabling dynamic study of lymphocytes within live pancreas tissue [49].

In other autoimmune diseases, the ability to isolate B cells from both the peripheral blood and disease target tissue has led to fascinating insights [50, 51]. However, relatively few studies have examined antigen-specific BCRs in human type 1 diabetes, with existing studies focused primarily on phenotypic changes among insulin-binding B cells [52–54]. As with T cells, this is due to difficulties sampling from the pancreas and low frequencies of the clones of interest in the peripheral blood. BCR sequencing studies in individuals with type 1 diabetes identified a skewed frequency of *BCR heavy chain* (also known as immunoglobulin heavy chain or *IGH*) V gene occurrence in pancreatic draining lymph nodes relative to control individuals [55]. The specificity of these BCRs was, however, not determined in this bulk BCR sequencing study, as paired heavy and light chain sequences were not generated. Until recently, there were no methods available equivalent to T cell AIM assays and tetramer tools. However, technical developments such as dCODE Klickmers [56] and LIBRA-seq (LInking B cell Receptor to Antigen specificity through SEQuencing [57, 58]), which link single-cell BCR sequencing to antigen specificity and gene expression information, now offer the possibility of enriching diabetes autoantigen-specific B cells from the peripheral blood and facilitating epitope mapping of autoreactive B cell responses.

## TCR databases with antigen/epitope specificity

A number of databases exist that contain sequencing information on T cells with known (auto)antigen specificity. These include VDJdb [59] (~92,000 TCR sequences), McPAS [60] (~40,000 TCR sequences) and the Immune Epitope Database (IEDB) [61] (~185,000 TCR sequences). These databases curate data from papers that use TCR specificity assays to study TCR–epitope interaction and store this information with relevant metadata about the TCR, epitope and MHC presentation of that epitope. Although these are valuable resources, with less than 10^6^ TCRs, they only cover a small fraction of the possible number of receptors (estimated to be 10^19^) [62]. More problematic for the study of type 1 diabetes, only a fraction of these TCRs are islet antigen-specific and are therefore relevant to the study of the disease (McPas: ~1500; VDJdb: 12; IEDB: ~1700). In contrast, the iReceptor platform searches AIRR-seq data in the ADC, but the ADC does not currently contain antigen/epitope specificity. The iReceptor Gateway currently queries IEDB to determine antigen/epitope specificity [3]. Until the creation of the T1D TCR/BCR Repository (The Type 1 Diabetes T Cell Receptor and B Cell Receptor Repository), there was no type 1 diabetes data in the ADC.

## About the iReceptor platform

**Fig. 1:**
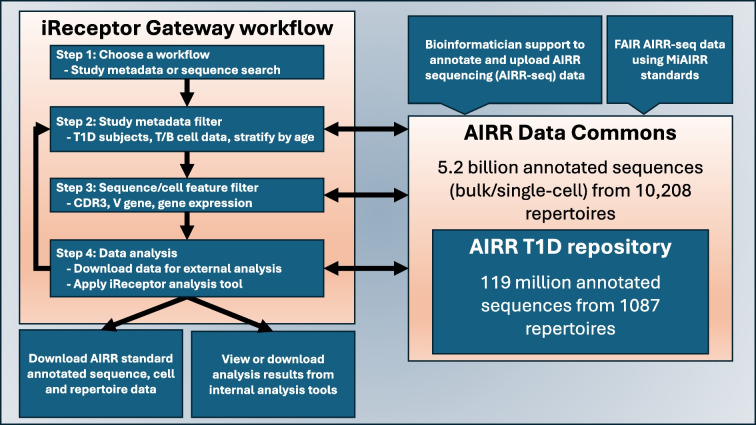
Overview of how the AIRR type 1 diabetes repository sits within the AIRR Data Commons and is accessed through the iReceptor Gateway. T1D, type 1 diabetes

**Fig. 2:**
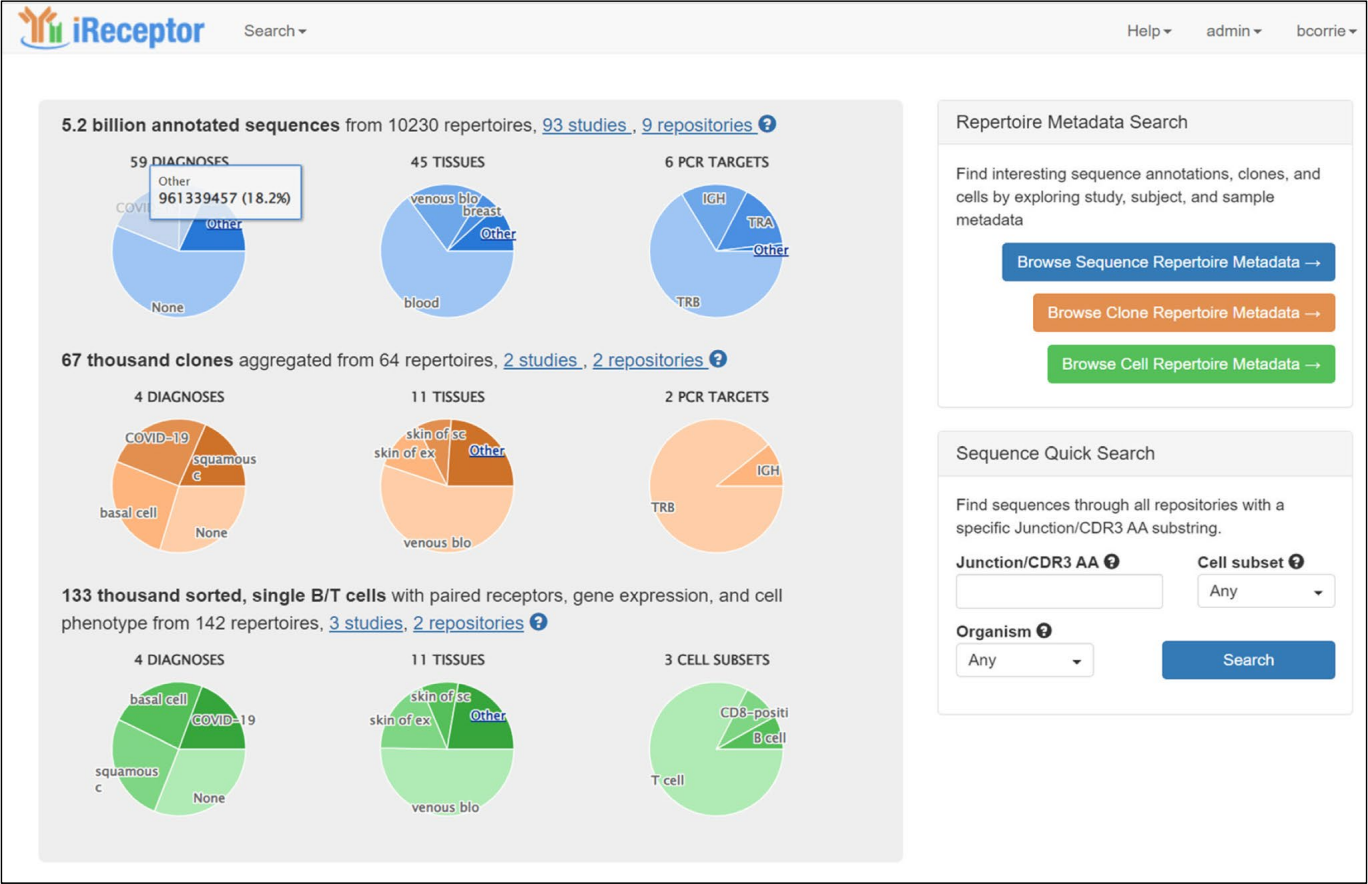
iReceptor Gateway overview page of the data in the ADC. Summary statistics of the annotated sequence, clone and single-cell data available in the ADC are provided. Users can select a ‘Repertoire Metadata Search’ or a ‘Sequence Quick Search’ workflow. The metadata search workflow can be done from the perspective of annotated sequences, clones or single-cell data. This is the user interface for Step 1 in the iReceptor Gateway workflow from Fig. 1. Reproduced with the permission of the iReceptor project

**Fig. 3:**
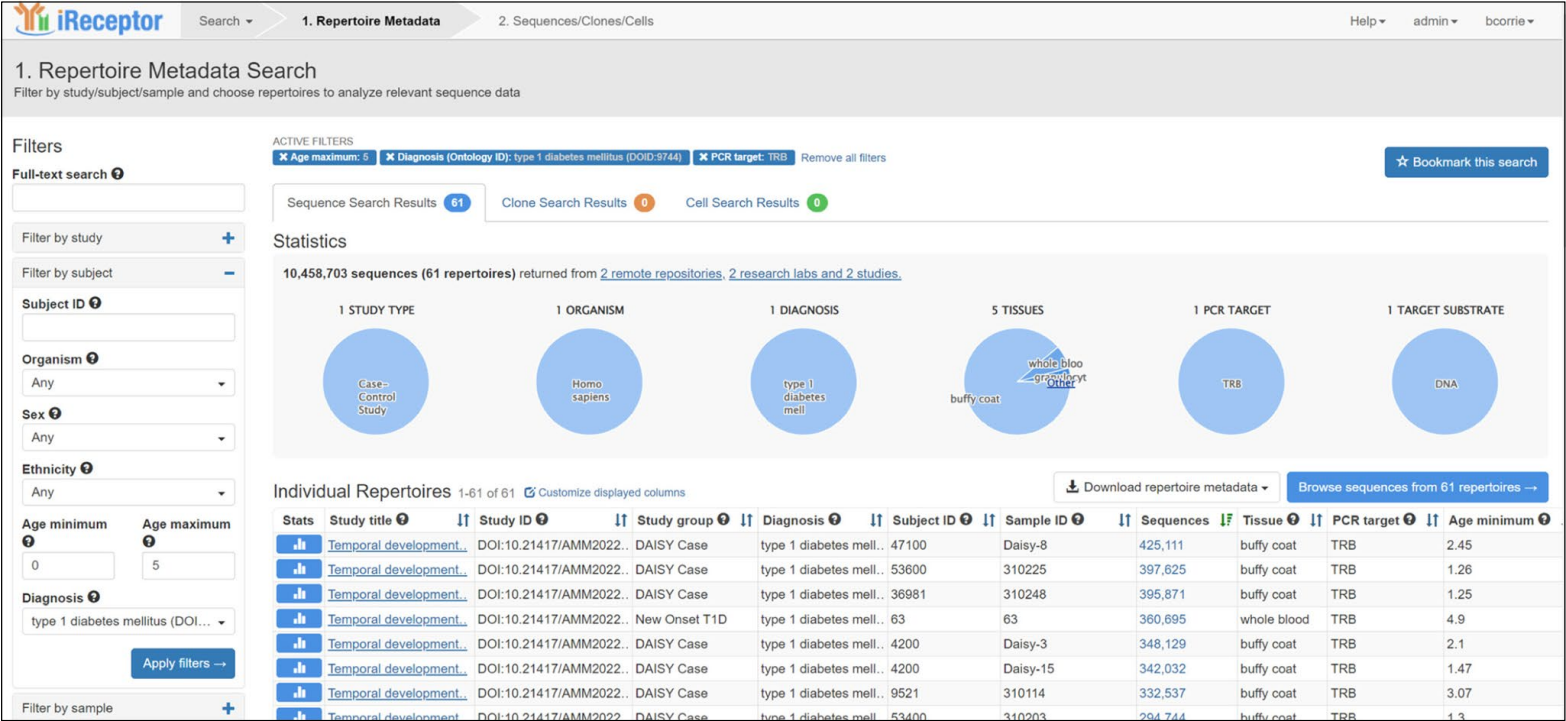
iReceptor Gateway ‘Repertoire Metadata Search’ page for annotated sequence data. Repertoires are filtered so that the data are limited to TCRβ sequences from individuals with type 1 diabetes. The data are further stratified to select only data from individuals between 0 and 5 years of age. Over 10 million annotated sequences from 61 repertoires and two studies in the ADC meet this search criteria. All of these type 1 diabetes data are retrieved from the AIRR T1D TCR/BCR Repository. This is the user interface for Step 2 in the iReceptor Gateway workflow from Fig. 1. Reproduced with the permission of the iReceptor project. TRB, alternative name for TCRβ

**Fig. 4:**
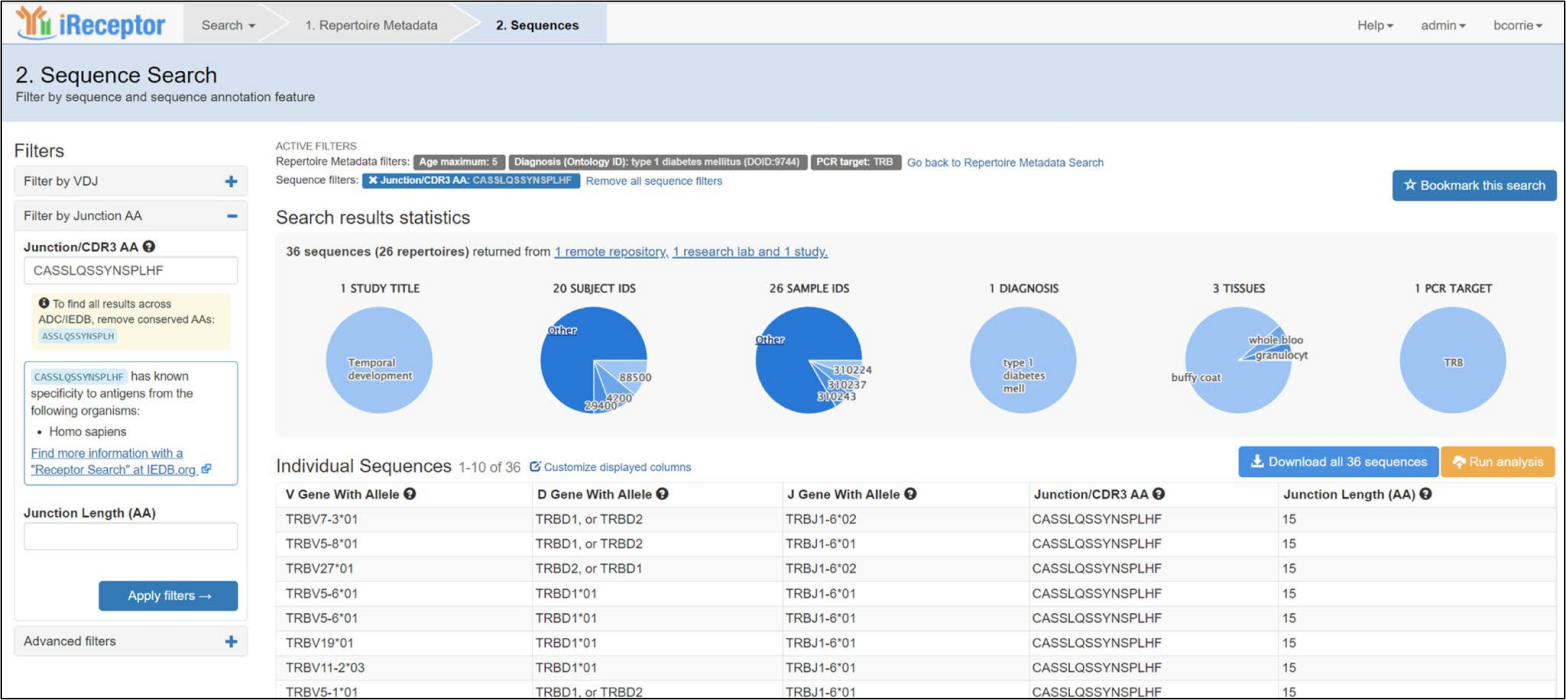
iReceptor Gateway ‘Sequence Search’ page. A search for a CDR3 amino acid sequence of interest (CASSLQSSYNSPLHF) is performed on the TCRβ data from an individual with type 1 diabetes between 0 and 5 years of age in the ADC. The iReceptor Gateway reports that it found 36 such sequences across 26 repertoires and 20 subjects, suggesting that this is a ‘public’ CDR3. It also reports that this CDR3 has a known antigen/epitope specificity and provides a link to external resources (e.g. IEDB) to find out further information about the antigen/epitope. At this stage, the user can either download the data for offline analysis or perform internally supported analyses of these data. This is the user interface for Step 3 in the iReceptor Gateway workflow from Fig. 1. Reproduced with the permission of the iReceptor project. TRB, alternative name for TCRβ

**Fig. 5:**
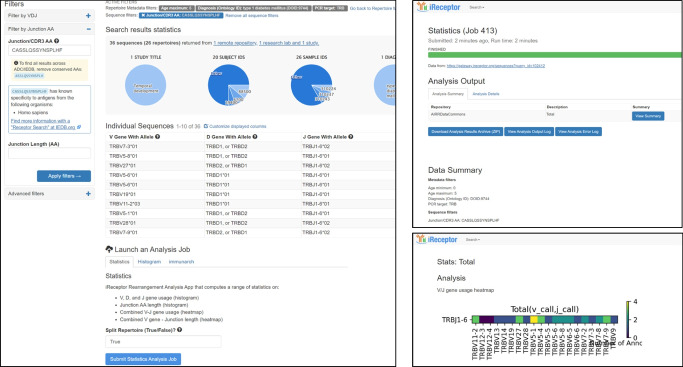
iReceptor internal statistical analysis of the selected data. The left frame is a view of a subregion of the ‘Sequence Search’ page, showing the set of analysis tools that are available for the selected data. The top right frame is the output of the ‘Statistics’ analysis tool. The user can either download the analysis results or view a summary of the analysis output. The search parameters for the repertoire metadata as well as the sequence features that were used to select the analysed data are displayed. The bottom right frame is a view of a subset of the analysis results, in this case a V/J gene heatmap for the CDR3 of interest. The results of this analysis provide the frequency for all TCRβ receptors (V/J gene combinations) for the CDR3 sequence CASSLQSSYNSPLHF from individuals with type 1 diabetes between 0 and 5 years of age in the ADC. This is the user interface for Step 4 in the iReceptor Gateway workflow from Fig. 1. Reproduced with the permission of the iReceptor project. TRB, alternative name for TCRβ

The iReceptor platform [3] (https://gateway.ireceptor.org) is based on FAIR (Findable, Accessible, Interoperable, Reusable) data principles [63] and allows access to curated AIRR-seq data and associated metadata in the ADC [4]. The overarching goal of the iReceptor platform is to enable researchers to increase the value of their data through sharing with the community. The ADC is an internationally distributed set of repositories that adhere to standards for sharing and analysing AIRR-seq data [64, 65] developed by the AIRR Community [66]. The ADC currently consists of over 5 billion annotated BCR/TCR sequences from more than 90 studies (Figs 1, 2). Figures 2, 3, 4 and 5 provide a detailed example of how to search the type 1 diabetes repository in the ADC through iReceptor.

What sets the iReceptor platform apart from other AIRR-seq (TCR/BCR) platforms is the ability to search extensive metadata (study, de-identified subject, sample, disease, tissues etc.) and AIRR-seq features (BCR/TCR annotations, cell clonal lineages, cell phenotype and cell–antigen reactivity) across all of the studies and all of the repositories in the ADC. In addition to federating the repositories in the ADC, iReceptor also links to external repositories such as NCBI to allow access to source sequence data, IEDB for immune receptor–antigen specificity [61], ArrayExpress/Gene Expression Omnibus (GEO) for gene expression [67], and Ensembl for definitions of expressed genes [68, 69]. Through this integration, iReceptor allows researchers to find relevant data that will help answer complex research questions at a scale and complexity that is challenging to achieve otherwise. For example, researchers can search for AIRR-seq data with specific characteristics at various levels, including study (e.g. methodology), subject (e.g. age, disease state, HLA genotype), sample (e.g. tissue type), and sample processing (e.g. cell phenotype) (Fig. 3).

In type 1 diabetes, the HLA type is particularly relevant, given the associations between TCRs and HLA and the high degree of HLA association with type 1 diabetes [1, 70]. Searches that filter samples on the basis of these metadata narrow the scope of data being considered from billions of records to a smaller subset of records that are more relevant to the research question and more amenable to detailed analysis. These filtered data can then be searched for BCR/TCR (e.g. CDR3, VDJ annotations) and cell characteristics (e.g. paired chains, expressed genes, cell reactivity) (Fig. 4). Once relevant data are found, the data can then be either downloaded by the researcher for analysis in their custom analysis pipelines or submitted to one of the analysis pipelines built into the iReceptor platform (e.g. CoNGA [71] or CellTypist [72]) (Fig. 5).

## Goals of the T1D AIRR Consortium

In a collaboration led by the A. Michels lab (University of Colorado), the T. Brusko lab (University of Florida), the iReceptor team (Simon Fraser University), the Human Pancreas Analysis Program (HPAP, [18, 72]), and the(sugar)science group (https://thesugarscience.org/), the aim of the T1D AIRR Consortium is to bring together type 1 diabetes-related AIRR-seq datasets into the T1D TCR/BCR Repository that are freely available, searchable and downloadable as a complete dataset for analysis in researchers’ pipeline of choice (e.g. R or Python). In order to provide data that are ML and artificial intelligence (AI) ready, type 1 diabetes datasets in the ADC, accessed through iReceptor, are being curated with optional, customised metadata fields including HLA typing, presence of AAb markers, immunoglobulin and TCR germline gene polymorphisms, disease progression, immune cell source, detailed protocol information for enrichment (e.g. AIM assays or tetramer sorting), gene expression (GEX) data and antigen specificity. It will encompass state-of-the-art scRNA-seq datasets generated by individual investigators, large-scale TCR sequencing projects (e.g. HPAP [18, 73]), and historical datasets [55].

The AIRR Community, including members of the iReceptor team, has published a general methods paper for sharing and reusing AIRR-seq data [74]. Our type 1 diabetes initiative extends this methodology to focus on identifying and onboarding type 1 diabetes research groups with the process of data curation in the ADC. Rigorous data curation that maximises the value of research data is costly and time consuming (see Note 4, page 469 in [74]). One goal of the T1D AIRR Consortium is to increase the capacity to share data. This is primarily accomplished through standardising study and data processing protocols such that data curation into the T1D TCR/BCR Repository is part of the general practice in the labs of the T1D AIRR Consortium members. In our experience, data curation is initially most efficient when study, domain, bioinformatics and technology experts work together to establish a curation pipeline for a research group, but once that pipeline is established the incremental cost of curation to maximise data reuse is greatly reduced. Each subsequent use of the curation pipeline increases its efficiency, making the data curation process easier for the research group. Type 1 diabetes researchers may also wish to include depositing data in the ADC as part of their data management plans in grant applications, as a straightforward way of fulfilling their FAIR data obligations. This is in fact already happening for the HPAP bulk TCR data, with plans to follow for the bulk BCR and single-cell data.

**Fig. 6:**
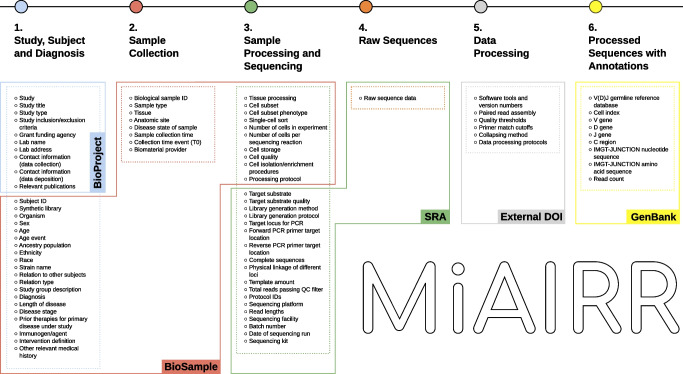
An overview of the six MiARR [64] standard classes of data and associated data fields. More details on the standard are available at the AIRR Community website: https://docs.airr-community.org/en/stable/standards/overview.html. Copyright AIRR Community, reproduced under a CC BY 4.0 licence. QC, quality control

The Minimal information about AIRR (MiAIRR) standard [64] was developed by the AIRR Community as a guide to the documentation and curation of studies that involve AIRR-seq data and is the standard that is used to describe study metadata in the ADC. Up to date information, including detailed descriptions of the MiAIRR fields (Fig. 6), can be found on the AIRR Community documentation website (https://docs.airr-community.org/en/stable/standards/overview.html). In brief, MiAIRR consists of several classes of metadata:

1. **Study, subject and diagnosis**: describes the study (e.g. title, research group, funding, publications), the study methodology used, the subjects in the study (species, age, sex), genotype characteristics (HLA, BCR and TCR genotype), and clinical characteristics (AAb status, disease diagnosis and duration, vaccinations, treatments such as immunotherapies).
2. **Sample collection:** describes the sample collection process used, tissue (tissue type, anatomical location of sample), disease state of tissue, sample collection timepoints, and details of any enrichment strategies such as tetramer sorting.
3. **Sample processing and sequencing**: describes how the samples were processed in preparation for sequencing and how that sequencing was performed, including sample processing, cell processing, library preparation and the sequencing process (e.g. sequencing platform, sequencing facility, sequencing date).
4. **Raw sequences**: includes Sequence Read Archive (SRA) data.
5. **Data processing**: describes how the sequenced data were processed to produce the AIRR-seq related digital artefacts that are stored in the ADC (annotated BCR/TCR sequences, cells, cell expression, cell reactivity), including quality control processing, primer processing, software packages and versions used, and other data processing protocols.
6. **Processed sequences with annotations**: includes VDJ germline reference databases, V(D)J gene segment calls, IMGT nucleotide and amino acid sequences (www.imgt.org/), and read counts.

Although the MiAIRR standard contains a large number of fields, it does not require that all studies must capture all fields. Rather, it should be considered a guide as to what a research group might want to include in the planning and curation of a study. It is worth noting that, even though a metadata field might not be considered important to answer an investigator’s specific research question, the field might be important for others to reuse those data. The AIRR Community recommends that as many of the MiAIRR fields be populated as possible in order to maximise data reuse.

## Onboarding data

The T1D AIRR Consortium is now a solid community of collaborators who champion the benefit of FAIR research data. Led by the(sugar)science team, outreach to the type 1 diabetes community has resulted in steady growth of consortium members. Each collaborator researching type 1 diabetes who has expressed a willingness to share data is contacted by the data curation team to establish scientific and bioinformatics expertise within the study team. This process of proactive collaboration is critical to the efficient initial curation of study metadata for that research group. Example study metadata worksheets are provided, using the AIRR Community standards, as a basis for this curation, and the study domain experts work closely with the iReceptor curation team to establish a rigorous set of metadata, including type 1 diabetes-specific metadata elements, for each study. This is an iterative process, where the study metadata are refined over time. Once established, this curation pipeline is followed for subsequent studies. Current and new studies use the study metadata curation pipeline up front as part of the planning and study execution process, making it straightforward and efficient to curate new studies in the ADC.

The iReceptor curation team also works with the study bioinformatician to prepare the annotated AIRR-seq or scRNA-seq data for loading. Although the iReceptor platform can load the output of many of the standard VDJ and single-cell annotation tool chains (e.g. IgBLAST [www.ncbi.nlm.nih.gov/igblast/], MiXCR [https://mixcr.com/], 10X CellRanger [www.10xgenomics.com/support/software/cell-ranger/latest], Adaptive ImmuneAccess [https://clients.adaptivebiotech.com/immuneaccess]), it is sometimes necessary to transform these data as part of this preparation.

It is important to note that the key benefit of including the data in the ADC is the value-added curation of standards-based AIRR-seq and scRNA-seq data and their associated metadata, which enables data reuse. However, this does not preclude the necessity of maintaining the raw sequence data in a standard sequence archive such as the International Nucleotide Sequence Database Collaboration repositories (INSDC; www.insdc.org/), European Nucleotide Archive (ENA; www.ebi.ac.uk/ena/browser/home) [75], SRA (www.ncbi.nlm.nih.gov/sra), database of Genotypes and Phenotypes (dbGaP; www.ncbi.nlm.nih.gov/gap/), ArrayExpress (www.ebi.ac.uk/biostudies/arrayexpress) or GEO (https://www.ncbi.nlm.nih.gov/geo/).

The ADC has been established as an international resource, with repositories currently spanning Canada, the USA and Germany. The AIRR Community encourages researchers to curate their own data at their home institution, and software has been developed that enables this through the download and installation of the open source iReceptor Turnkey software (https://github.com/sfu-ireceptor/turnkey-service-php). Research groups with large amounts of AIRR-seq data are encouraged to consider running a repository of their own to curate and share their data. The iReceptor team uses the iReceptor Turnkey to run several ADC repositories, including the iReceptor Public Archive (IPA), a COVID-19 repository, as well as other repositories in collaboration with other researchers.

As part of the T1D AIRR Consortium, the iReceptor team runs the T1D TCR/BCR Repository. Once the study metadata and the sequencing data have been prepared for a study from a group in the consortium, the iReceptor curation team loads the data and study metadata into the T1D TCR/BCR Repository. Although documentation is available for loading data into the iReceptor Turnkey (https://github.com/sfu-ireceptor/turnkey-service-php#loading-your-own-data), the iReceptor team performs this role on behalf of collaborators in the consortium. The iReceptor team also supports the repository operationally to ensure reliability. Once the data are loaded, the repository is put into production, and the data are then available to be queried, analysed or downloaded as part of the ADC by any user of the iReceptor platform.

**Fig. 7:**
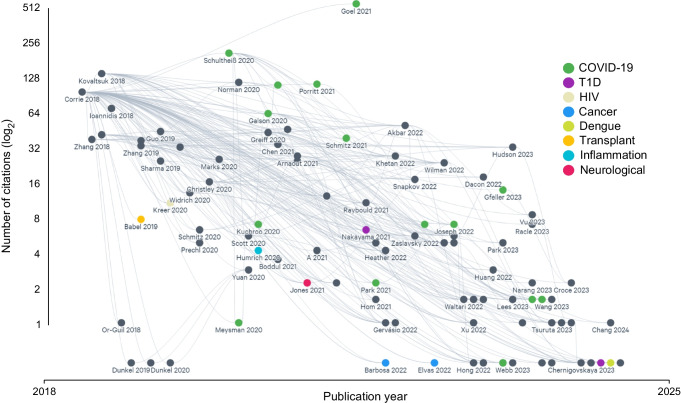
Citations as a metric of iReceptor manuscript influence, by disease. Citations of the iReceptor paper (Corrie et al 2018 [3], top left corner), coloured by high level disease classification. Grey citations are not disease specific and either represent standards (e.g. AIRR Standards), tools (e.g. ML), or techniques (e.g. repertoire classification) papers. Grey connecting lines indicate manuscripts that were cited in subsequent publications. Interactive visualisation of citation data is available at https://app.litmaps.com/shared/12ead8ba-35d9-4232-91a0-26da9e3c3f59; colour coding in the interactive visualisation is different from the figure above. T1D, type 1 diabetes

The impact of sharing AIRR-seq data and in particular, the impact of the iReceptor platform in facilitating this data sharing can be demonstrated by the fact that the iReceptor paper [3] has been cited over 100 times in the literature as a source to store, find or reuse AIRR-seq data (Fig. 7).

The first citation of data from the T1D TCR/BCR Repository has just been published, where data from the type 1 diabetes study from Seay et al [55] (loaded into the repository in March 2023) was reused in a recent cancer study from Sammut et al [76] that explored BCR clonal centrality in immunosurveillance across cancer, type 1 diabetes and multiple sclerosis.

## Use cases

### Gaining insight into type 1 diabetes

The overarching aim of the T1D TCR/BCR Repository is to enable development of strategies to track immune repertoire changes that predict type 1 diabetes progression risk and/or individual response to immunotherapy. In order to achieve this, it is important to be able to reach a critical mass of type 1 diabetes data that is available to the research community. To date, six studies have been curated by the T1D AIRR Consortium into the T1D TCR/BCR Repository and are now available as part of the ADC, consisting of over 110 million annotated sequences from 366 individuals, eight tissue types and 12 T cell and B cell subsets. Table 2 provides an overview of publicly available type 1 diabetes repertoire datasets in the ADC.

**Table 2:** Repertoire data from type 1 diabetes studies currently deposited in ADC by the T1D AIRR Consortium

Study	Cell and data type(s)	Tissue type(s)	Single-cell or bulk?	No. of sequences	Disease metadata available
Gomez-Tourino et al [47]	Central memory CD4^+^ T cells, true naive CD4^+^ T cells, CD4^+^ Treg, stem-cell like memory T cells (Tscm), CD4^+^ T cells (α/β TCR)	Blood	Bulk	13M T1D, 21M NT1D	Age, sex, race, diabetes duration
Mitchell et al [18]	T cells (TCRβ)	Blood	Bulk	46M T1D, 15M NT1D	Age, sex, ethnicity, diabetes duration, islet AAb, HLA class I and II types, longitudinal samples
Seay et al [55]	B cells (BCR heavy chain), CD4^+^ T cells, CD8^+^ T cells, CD4^+^ Treg (TCRβ)	Blood, irrelevant lymph node, islet of Langerhans, pLN, spleen	Bulk	5.2M T1D, 1.7M T2D, 3.6M NT1D	Age, diabetes duration, prior therapies, medical history, HLA, islet AAb
Anderson et al [38]	CD4^+^ T cells, CD8^+^T cells (α/β TCR)	Islet of Langerhans	Single-cell	5K T1D, 2K NT1D	Age, diabetes duration, prior therapies, medical history, HLA, islet AAb
Culina et al [77]	Central memory CD4^+^ T cells, central memory CD8^+^ T cells, effector memory CD8^+^ T cells, naive thymus-derived CD8^+^ T cells, naive thymus-derived CD4^+^ T cells (α/β TCR)	Blood	Bulk	4.3M T1D, 1.8M T1DR, 3.6M NT1D	Age, ethnicity, diabetes duration, HLA, islet AAb
HPAP T1D TCRβ data [73]	TCRβ	Spleen	Bulk	415K T1D, 107K T2D, 658K NT1D	Age, medical history, islet AAb

K, thousand; M, million; NT1D, control group without type 1 diabetes; T1D, type 1 diabetes; T1DR, type 1 diabetes risk; T2D, type 2 diabetes

Once such a critical mass is reached, researchers can reuse these data to explore new hypotheses, which hold potential to drive new developments in type 1 diabetes. For TCRs, this could include analysis of CDR3 length [47], motif sharing [78], modelling of repertoire changes during type 1 diabetes development [18] and during immunotherapy, prediction of TCR specificity [48], understanding the role of immune response to viruses in type 1 diabetes development [79], and understanding features of type 1 diabetes autoantigen-specific TCRs such as positioning of charged amino acids and hydrophobic residues within the CDR3 region. It has recently been demonstrated that specific diabetes autoantigen-specific TCRβ chains showed distinct patterns of accumulation during type 1 diabetes development, suggesting that they could be used as biomarkers [40] and an increase in validated type 1 diabetes-specific TCR sequences will expedite this process. Furthermore, the ADC contains many TCR repertoires from healthy control individuals, which allows immediate comparison of type 1 diabetes results with other repertoires (critical in determining the limits of public clonotypes) and will facilitate the understanding of the differences between people with and without type 1 diabetes.

BCRs in type 1 diabetes remain a largely untapped resource [34], but it is envisaged that identification of specific CDR3s may be additive when combined with islet AAb as predictive tools. The iReceptor platform enables deposition of linked antigen binding, single-cell BCR sequencing, gene and protein expression data types, which can be exported for analysis using immune repertoire tools such as the Immcantation suite of tools (including Change-O, Alakazam and SHazaM [80]) to identify immune repertoire features, including B cell V(D)J identities, CDR composition, clonotypes, isotypes, per cent somatic hypermutation, and BCR physiochemical properties (e.g. CDR charge, length, polarity). Several analysis packages exist that support gene expression and pathway analysis (e.g. Seurat [81], g:Profiler [82] and CytoScape [83]), which can be merged with immune repertoire analysis outputs. These additional data types can also be housed in the T1D iReceptor database, along with AIRR data.

### COVID-19 as an example of rapid data sharing that led to improved understanding of immune contributions to disease

We demonstrate the value of disease-specific repositories, data sharing and data reuse within the ADC that was catalysed during the COVID-19 pandemic [84] as an example of our vision for the type 1 diabetes community. Early in the COVID-19 pandemic, it was realised that the AIRR Community and, in particular, the ADC, were well positioned to have a significant impact on the understanding and treatment of COVID-19. Like in many areas, COVID-19 emphasised the need to share data, and in March 2020, the AIRR Community made a call for sharing of AIRR-seq data from consented COVID-19 patients (https://www.antibodysociety.org/airr-community/covid-19-demands-increased-public-sharing-of-biomedical-research-data/). In May of 2020, one of the first studies with publicly available AIRR-seq data was published as a pre-print [85], and by June 2020, the iReceptor team had provisioned an ADC COVID-19 repository and curated these data. This was rapidly followed by the curation of a total of 9 COVID-19 studies by the end of 2020. In addition, iReceptor housed data to evaluate SARS-CoV-2 vaccine responses, providing large numbers of spike-binding BCR sequence data to the community (for example [86]).

The impact of the availability of disease-specific data (COVID-19 in this case) for reuse by other researchers can be demonstrated by considering several use cases from the literature. For example, Heming et al [87] studied the immune cell profiles of cerebrospinal fluid from eight neuro-COVID patients, identifying expanded clonotypes in mild and severe neuro-COVID. They utilised iReceptor to discover subjects diagnosed with COVID-19 from seven studies in the ADC. They retrieved >180 million CDR3 amino acid sequences from COVID-19 patients in the ADC and determined the overlap of CDR3 amino acid sequences from their own data, identifying SARS-CoV-2-specific clones from cerebrospinal fluid. They also used TCR sequences from healthy control individuals in the ADC and by comparing the repertoires demonstrated that TCRs were shared between the cerebrospinal fluid and periphery in COVID-19.

It should be noted that the key feature of this analysis is the CDR3 amino acid sequence, which is a fundamental part of the value-added annotation that the ADC provides. If these data were not available in this form from the ADC**,** the raw sequence data from all the studies listed above would have had to be re-annotated at enormous cost in terms of time, human effort and computational resources. Through the iReceptor platform, the researchers were easily able to find and access relevant data (from individuals both with and without COVID-19), download that data for analysis and comparison with their own data, and directly reuse the identified CDR3 regions in these data for their analysis. Similarly, a paper by Schultheiß et al published one of the early studies on COVID-19 that tracked disease progression over time [2]. The authors worked with the iReceptor team to ensure the data were available in the ADC when the paper was published. This paper is highly cited, and currently, 15 publications cite both the iReceptor paper [3] and the Schultheiß paper, indicating the likely reuse of the Schultheiß data from the ADC.

More recently, we have seen extensive citations of iReceptor from papers discussing the value of the curated and annotated data in the ADC for training and assessing ML algorithms [88]. The power of the iReceptor platform in conjunction with ML approaches is also demonstrated in the COVID-19 arena. Park et al used iReceptor to obtain TCR datasets from individuals with COVID-19 [89]. They then used both standard analysis tools, such as Immunarch [90], OLGA [91], and GLIPH2 [92], as well as a variety of k-mer-based ML models, to gain insight into the TCR repertoires. They identified features of COVID-19-specific TCRs such as gene usage and motif. Moreover, the ML approaches could identify not only whether a person had COVID-19 but also the disease severity, based solely on TCR repertoire features.

COVID-19 BCR datasets using iReceptor have been similarly used to compare and validate protein language models that predict BCR specificity [93] and recognise COVID-19-specific BCR signatures [94]. Use of iReceptor to understand BCR repertoires was demonstrated by Waltari et al, who accessed datasets through iReceptor to develop AIRRscape, an R Shiny-based app and website to visualise BCR repertoires [95]. They downloaded bulk repertoire BCR datasets from COVID-19-infected individuals from iReceptor and complemented them with datasets of known monoclonal antibodies. They demonstrated features of the bulk repertoires that differed between the distinct infections, such as convergent motifs and V-J pairings.

The iReceptor team was able to work directly with many of the COVID-19 researchers to curate and make these data available. It is this model of data sharing that the T1D AIRR Consortium will emulate and promote.

## Future collaborations and efforts

Looking ahead, the T1D AIRR Consortium will focus both on uploading historical type 1 diabetes datasets and enabling the type 1 diabetes community to upload contemporaneous datasets. Outreach through efforts of the(sugar)science will continue to recruit scientists from the type 1 diabetes community to join the T1D AIRR Consortium, enabling continual growth of studies in the T1D TCR/BCR Repository from a range of research groups. The T1D AIRR Consortium encourages researchers to share their valuable type 1 diabetes data and welcomes new collaborators to the consortium. To learn more and join the monthly consortium working group, contact: T1DAIRR@thesugarscience.org

## Data Availability

All data in Table 2 are available from https://gateway.ireceptor.org/login Data from Fig. 7 are available at https://app.litmaps.com/shared/12ead8ba-35d9-4232-91a0-26da9e3c3f59

## Abbreviations

AAb: Autoantibody/autoantibodies
ADC: AIRR Data Commons
AIM: Activation-induced marker
AIRR: Adaptive Immune Receptor Repertoire
AIRR-seq: AIRR sequencing
BCR: B cell receptor
CDR3: Complementarity determining region 3
FAIR: Findable, Accessible, Interoperable, Reusable
GEO: Gene Expression Omnibus
HPAP: Human Pancreas Analysis Program
IEDB: Immune Epitope Database
MiAIRR: Minimal information about AIRR
ML: Machine learning
pLN: Pancreatic lymph node(s)
SARS-CoV-2: Severe acute respiratory syndrome coronavirus 2
scRNA-seq: Single-cell RNA-seq
SRA: Sequence Read Archive
T1D TCR/BCR Repository: The Type 1 Diabetes T Cell Receptor and B Cell Receptor Repository
TCR: T cell receptor
TCRβ: T cell receptor β chain
Tfh: T follicular helper
Treg: Regulatory T cell(s)
VDJ: Variable, diversity and joining gene segments
